# Analysis of Participation Patterns and Injury-Triggering Factors in Elderly Grassroots Sports Using Association Rule Mining

**DOI:** 10.3390/life16030467

**Published:** 2026-03-12

**Authors:** So Yoon Lee

**Affiliations:** Department of Sport Senior, Korea National Sport University, Seoul 05541, Republic of Korea; joyful0202@knau.ac.kr; Tel.: +82-2-410-6931

**Keywords:** elderly grassroots sports, sports injury, association rule analysis, data mining, sports safety

## Abstract

This study aimed to identify structural relationships between sports participation patterns and injury risk factors among older adults using data mining techniques, addressing limitations of prior descriptive research. Data from the 2024 Sports Safety Accident Survey were analyzed, including 352 adults aged 65 years and older. Eight key variables related to participation and injury were examined using association rule analysis with the Apriori algorithm. High-risk injury patterns were associated with irregular participation in non-sport-specific locations during late-night hours and with excessive movement during prolonged, daily participation, particularly in the morning. Injuries most frequently affected major joints of the upper and lower limbs. Sports injuries in older adults arise from complex interactions among temporal, environmental, and behavioral factors. These findings support the development of targeted safety guidelines and injury prevention strategies tailored to participation patterns in the aging population.

## 1. Introduction

The rapid aging of the global population structure presents a critical challenge to healthcare systems and socioeconomic sustainability. South Korea became an aged society in 2018, with the population aged 65 and older exceeding 14% [1], and is currently on the verge of transitioning into a super-aged society by 2026. While the remarkable advancement of modern medicine has extended life expectancy, this “external extension of survival” does not inherently guarantee an improved quality of life. Consequently, the paradigm of contemporary gerontology has shifted from mere survival to “successful aging”, which emphasizes maintaining physical and mental functions to lead a socially dynamic life.

In this context, grassroots sports serve as a powerful protective factor that enhances self-esteem and promotes successful aging among the elderly [2]. National surveys on physical activity participation reveal a sharp increase in the participation rate of elderly people, rising from 45.5% in 2017 to 62.2% in 2018. This suggests that grassroots sports have established themselves as a core non-pharmacological intervention for alleviating geriatric depression, maintaining cognitive function, and preventing cardiovascular diseases [3,4].

However, the expansion of physical activity participation inevitably carries the risk of sports-related accidents and injuries, creating a critical paradox in geriatric health. Degenerative musculoskeletal changes and diminished balance associated with aging can lead to severe physical damage from even minor impacts [5]. Unlike younger populations, the elderly face a higher risk of these injuries transitioning into permanent loss of Activities of Daily Living (ADLs) or chronic physical disabilities. Notably, injury experiences can trigger kinesiophobia (fear of movement), accelerating withdrawal from physical activities and forming a “vicious cycle of injury” that co-occurs with frailty and secondary diseases.

Despite these risks, existing research on sports injuries in the elderly has predominantly focused on descriptive frequency reports, the revitalization of injury-related services [6,7,8], or cognitive domains and satisfaction levels [9,10]. There is a notable lack of research investigating the complex interplay between exercise intensity, frequency, and injury causes specifically within the elderly population. Consequently, there is a pressing need for a new analytical approach based on data mining to identify non-linear accident scenarios and latent risk patterns—elements that are difficult to capture through traditional linear statistical methods that assume variable independence. Therefore, this study applies Association Rule Analysis (ARA) to explore the non-linear structural relationships between elderly sports participation patterns and injury-triggering factors.

Furthermore, integrating 4th Industrial Revolution data science technologies into health policy is essential for ensuring empirical policy utility. While existing fragmented safety guidelines fail to reflect the diverse activity patterns of individual seniors, Association Rule Analysis using large-scale public data enables precise risk prediction by capturing hidden correlations in “low-frequency, high-risk” accidents. This provides a strategic foundation for preemptively structuring potential accident scenarios and concentrating limited national budgets on the points of highest risk. Ultimately, this study aims to establish a qualitative safety net commensurate with the quantitative expansion of grassroots sports, thereby serving as a policy turning point in which elderly sports participation functions as a driver for extending healthy life expectancy rather than a factor threatening national medical finances.

## 2. Method

### 2.1. Materials and Methods

To investigate the status of injuries and the structural characteristics of participation patterns among elderly grassroots sports participants, this study utilized data from the ‘Sports Safety Accident Survey’ conducted by the Korea Sports Safety Foundation (KSSF). As of October 2024, the survey population was defined as sports participants across 64 national sports organizations registered in the ‘Sports Support Portal’ of the Korean Sport & Olympic Committee. For the purpose of this study, data were specifically extracted for older adults aged 65 and over from the survey records collected between 1 November and 14 December 2024. This study is a secondary data analysis utilizing public data provided by the KSSF, a public institution (https://www.sportsafety.or.kr).

Based on the reliability of the raw data, a total of 352 participants who had experienced sports-related injuries were selected as the final subjects for analysis. This study specifically focused on this injured cohort to examine the structural associations and co-occurrence patterns of factors that lead to actual injury incidents. Consequently, the Association Rule Analysis (ARA) was conducted exclusively on these 352 cases, ensuring that the derived metrics—Support, Confidence, and Lift—reflect the internal mechanisms and behavioral configurations specific to injury occurrences. The sampling and data collection processes complied with the procedures for nationally approved statistics. The researcher reprocessed the publicly available data in accordance with the study’s objectives to perform the subsequent analyses.

### 2.2. Selection and Content of Survey Variables

To achieve the objectives of this study, an expert panel of six specialists was formed, consisting of three professors majoring in elderly physical education and three Ph.D. holders specializing in measurement and evaluation. Through expert meetings, variables were selected from the raw items of the Korea Sports Safety Foundation survey (KSSF), a nationwide secondary dataset systematically designed by public health and sports safety experts to monitor injury patterns. During the selection process, content validity was ensured by comprehensively considering the modalities of elderly sports participation and injury-related factors. The expert panel focused on ensuring that the selected variables reflect the multidimensional risk factors commonly recognized in injury prevention literature. Specifically, the structure of the KSSF survey inherently aligns with the fundamental epidemiological framework that categorizes injury risks into host factors, exposure patterns, and environmental contexts. This resulted in the final determination of eight variables that capture the core dimensions of injury risk.

The selected survey variables were structured into three domains: demographic characteristics, participation behavioral characteristics, and injury-related characteristics. First, ‘Gender’ was included to identify the intrinsic host information of the subjects. Second, to examine the extrinsic exposure patterns of sports participation, ‘Frequency of Activity’, ‘Duration of Activity’, ‘Time of Activity’, and ‘Place of Activity’ were established as variables, representing the temporal and spatial context of participation. Third, to identify the actual status of injuries and their situational mechanisms, ‘Injury Experience’, ‘Injury Site’, and ‘Cause of Injury’ were selected. All variable names and sub-factors were applied identically to the definitions established by the Korea Sports Safety Foundation. The specific details of the survey variables finally determined and utilized for analysis in this study are presented in Table 1.

### 2.3. Data Analysis

The data processing methods for this study are as follows. First, to restructure the collected secondary data in accordance with the research objectives, Excel 2024 (Microsoft, Redmond, WA, USA) was utilized for data preprocessing and coding. Frequency Analysis was conducted using SPSS 26.0 (IBM, New York, NY, USA) to identify the general characteristics and injury status of elderly grassroots sports participants. Second, ARA based on Python 3.10 (Python Software Foundation, Wilmington, DE, USA) was applied to investigate the structural associations between physical activity patterns and factors associated with injuries. The Apriori algorithm was employed to extract frequent item-sets. While this method identifies non-random co-occurrence patterns, it should be noted that the derived rules represent associative relationships—such as the “Sports Paradox” involving high-risk configurations—rather than direct causal mechanisms. Accordingly, correlational terms like “co-occurrence” are used throughout the analysis to clarify ARA’s associative nature. Third, three metrics—Support, Confidence, and Lift—were calculated to verify the validity of the association rules. In this study, the minimum support threshold was specifically set at 0.01. This strategic setting was necessary to capture “niche patterns” within the injured sample (N = 352), as a higher threshold would risk filtering out critical, high-risk injury configurations that possess high clinical and safety significance. Given the sample size, a support of 0.013 corresponds to approximately 4–5 cases; these are interpreted as exploratory subgroup findings rather than generalized trends. To ensure the rigor and stability of these rules, a sensitivity analysis was performed by comparing results across different support thresholds (e.g., 0.01 vs. 0.02). The consistency in the extracted rules confirmed their stability, addressing potential critiques regarding small-sample variance. Furthermore, the absolute case numbers for each rule were monitored alongside the metrics to account for small cell counts and to avoid over-interpreting spurious associations. Among the derived rules, only patterns with a Lift value exceeding 1.00 were selected, indicating a positive association beyond mere chance. The formulas for calculating Support, Confidence, and Lift are as follows (Equation (1)).

(1) $$Support(A,B)=P(A\cap B)\;Confidence\left(A\to B\right)=\frac{P\left(A\cap B\right)}{P(A)}\;Lift=\frac{P(B|A)}{P(B)}=\frac{P(A\cap B)}{P(A)P(B)}=\frac{Confidence}{P(B)}$$

## 3. Results

### 3.1. Analysis of Participation Patterns Among Elderly Grassroots Sports Participants

#### 3.1.1. Comprehensive Analysis of Participation Patterns

Association rule mining was applied to identify the complex structure of elderly sports participation patterns. To ensure independence between variables, the top five patterns were extracted after excluding category overlaps (Table 2). First, the analysis revealed that activities during late-night hours were most strongly associated with irregular participation in non-sport-specific location, such as mountains or roads (Lift: 22.00). Second, when participants utilized Dedicated sports facilities (e.g., swimming pools or golf courses), activities lasting less than 3 h were characterized by a very low frequency of once every 4–5 months (Lift: 16.00). Third, the pattern with the next highest association showed that participation during morning and afternoon hours was closely correlated with regular participation of 2–3 times a week for less than 2 h (Lift: 12.80).

#### 3.1.2. Analysis of Participation Patterns by Gender

Association rule analysis was conducted to elucidate the complex structure of participation patterns among elderly males. To ensure independence between variables, the top five patterns were extracted after excluding category overlaps (Table 3). The results indicated that activities during morning and afternoon hours were most strongly associated with a regular participation pattern of 2–3 times a week for less than 2 h (Lift: 12.75). Second, participation during early morning and evening hours formed a very powerful correlation with short-term activities (less than 1 h) in non-sport-specific location such as mountains, roads, and parks (Lift: 12.36). Notably, this rule demonstrated a Confidence of 1.000, proving that participants during these specific time slots opted for short-term activities in non-sport-specific location without exception. Third, an extremely low participation frequency of once every 6 months was closely related to afternoon hours and the use of school playgrounds (Lift: 11.33).

Table 4 presents the extracted top five association rules characterizing the physical activity participation patterns of female older adults. The analysis reveals that the associative strength between variables is significantly more pronounced in the female group compared to their male counterparts. Specifically, a pattern of irregular participation with a duration of less than 30 min exhibited the highest degree of association across early morning, morning, and afternoon periods (Lift: 80.00). Similarly, activities occurring once a week for less than 3 h were distributed across morning, afternoon, and evening hours without temporal restriction (Lift: 80.00).

Notably, these rules achieved a maximum Confidence of 1.000, indicating a deterministic relationship where participants engaged in low-frequency, short-term activities utilize daytime and evening hours with high flexibility. To ensure the capture of such nuanced behavioral patterns within a sparse dataset, the minimum support threshold was strategically set at 0.01. While these high-lift associations (Lift: 80.00) are derived from a relatively small segment of the sample (Support = 0.013), the absolute confidence underscores the empirical reliability of these patterns within specific niche subgroups. Furthermore, activities conducted during early morning and night hours showed a perfect alignment with high-frequency participation (4–5 times a week) in non-sport-specific locations (Lift: 80.00), highlighting distinct environmental and temporal preferences among active female seniors.

### 3.2. Frequency and Pattern Analysis of Sports Injuries Among Older Adults

#### 3.2.1. Frequency Analysis of Injury Causes and Anatomical Sites

Table 5 presents the analysis of injury causes among elderly grassroots sports participants. The most prevalent cause was “excessive movement” exceeding one’s physical capabilities, accounting for 33.1% (52 cases) of the total. This was followed by “slipping and falling” (21.7%), “collision with another person” (9.6%), “tripping over an object” (8.9%), and “awkward landing after a jump” (7.0%). These findings confirm that factors related to diminished individual motor control and loss of balance are the primary mechanisms of injury, rather than external environmental factors.

Table 6 presents the results of the frequency analysis regarding injury sites. The findings indicate that the “shoulder” and “ankle” were the most frequently injured areas, with 11 cases (11.6%) each. These were followed by the “knee” and “elbow” at 9 cases (9.5%) each, and the “wrist” at 7 cases (7.4%). The top five injury sites are all concentrated in the major joints of the upper and lower extremities. In particular, the incidence of injuries was found to be similar between lower extremity joints (ankle, knee) and upper extremity joints (shoulder, elbow, wrist).

#### 3.2.2. Injury Patterns Among Elderly Grassroots Sports Participants

Table 7 presents the results of the injury pattern analysis among elderly grassroots sports participants. The findings identify excessive movement within dedicated sports facilities as a common cause of injury. Specifically, the risk of injury was most pronounced among the high-intensity participation group, who engage in activities daily for more than 3 h during the early morning (Lift: 7.438). Furthermore, the group participating 4–5 times a week during the afternoon also exhibited a powerful injury induction pattern associated with excessive movement in dedicated facilities, recording a Confidence of 1.000 (Lift: 2.833). This indicates a structural risk characteristic tied to specific activity time slots. Lastly, regular participants during the morning hours also showed a high correlation with injuries caused by excessive movement within dedicated facilities (Lift: 1.889).

## 4. Discussion

This study employed ARA to elucidate the structural relationships between the physical activity patterns of elderly grassroots sports participants and the multifaceted factors associated with injury. Based on the empirical findings, the primary points of discussion are as follows.

First, the analysis of activity patterns revealed distinct clustering based on temporal and spatial availability. Notably, the pattern characterized by “late-night activity in non-sport-specific locations (e.g., mountains or roads) with irregular participation” exhibited the highest lift. This identifies a critical blind spot in sports safety for the elderly. Despite the high level of safety consciousness generally reported among leisure sports participants [11], our findings suggest that informal activities conducted outside regulated facility hours are linked to higher vulnerability. To enhance policy utility, this finding suggests the implementation of time-based safety interventions. For instance, local municipalities could deploy automated mobile “safety alerts” or push notifications during late-night hours, specifically targeting regions with high informal activity, to remind elderly participants of risks associated with low visibility and unmanaged terrains.

Second, gender-based disparities in participation patterns were pronounced, with a significant difference in maximum Lift values. This gap indicates a higher degree of behavioral homogeneity within specific female subgroups. In ARA, such an exceptionally high Lift suggests that certain conditions—irregular and short-duration participation—are almost deterministically coupled with specific time-place configurations among female older adults. This reflects the “gendered time poverty” of the Korean elderly due to domestic chores and caregiving roles, women’s physical activity is intensely clustered within “interstitial” time slots [12]. Translating this into practice, we propose gender-responsive safety strategies. While men require education on acute injury prevention during high-intensity bursts, women would benefit more from gender-tailored warm-up protocols. These programs should focus on neuromuscular activation and joint stability to mitigate the chronic overuse injuries and fatigue co-occurring with their restricted and highly synchronized activity patterns.

Third, regarding injury etiology, the prevalence of “excessive movement” and “slips and falls” indicates that the primary mechanism of geriatric injury is the degradation of internal biomechanical control rather than external impacts [13,14]. The concentration of injuries in major joints—shoulders, ankles, and knees—suggests a compensatory kinematic chain: as lower-extremity stability is compromised, individuals rely on the upper extremities for protection [15]. This inherent vulnerability highlights the potential importance of lower-body resistance training as a fundamental prevention measure.

Fourth, the identified “Sports Paradox”—where “morning activity, daily for 3 h in dedicated facilities” shows a strong association with injury—offers profound implications. High-intensity activity performed before the physiological rhythm is fully activated, coupled with overuse fatigue, increases injury risk even in specialized environments like swimming pools or golf courses [16]. Safety education should therefore focus on the physiological necessity of prolonged warm-ups and the cognitive ability to modulate exercise intensity based on daily physical condition.

Furthermore, the methodological advantages of ARA compared to conventional linear models are evident. While regression analysis determines independent predictive power, ARA uncovers non-linear, multi-dimensional “if-then” scenarios without pre-defined hypotheses. This provides a holistic framework for understanding accident mechanisms—such as the “Sports Paradox”—that might be obscured in aggregate linear coefficients. However, ARA emphasizes associative strength through heuristic metrics like Lift rather than formal causal inference.

Finally, it is imperative to acknowledge the methodological characteristics and limitations. The ARA employed here identifies non-random co-occurrence patterns rather than direct causal mechanisms. To ensure transparency, we have reported the stability of these rules visualizing the relationship between support thresholds and absolute case numbers. While certain rules in the female group rely on relatively low support values (0.010–0.013, 4–5 cases), our sensitivity analysis—comparing results across thresholds of 0.01 and 0.02—demonstrated consistent rule stability. These findings should be viewed as exploratory evidence identifying “at-risk” subgroups. Future research incorporating longitudinal designs will be beneficial to further validate the long-term stability of the rare-event rules proposed in this study.

## 5. Conclusions

This study employed association rule analysis to elucidate the structural associations of injury risk underlying the rapid expansion of elderly grassroots sports. By analyzing the non-linear relationships within large-scale sports safety data, the following conclusions were reached.

First, elderly sports injuries are characterized by a multidimensional convergence of “Time–Facility–Behavior” rather than independent factors. Specifically, irregular activities in non-sport-specific location during late-night hours showed a high correlation with injuries, potentially reflecting environmental instability. Furthermore, gender-based analysis confirmed that while males show a higher co-occurrence with acute accidents within structured routines, females are more frequently associated with cumulative fatigue and overuse injuries stemming from flexible, distributed activity patterns throughout the day.

Second, the injury mechanism in the elderly is predominantly observed alongside deficiencies in individual biomechanical control rather than external impacts. The concentration of injuries—primarily co-occurring with excessive movement and slips—in major joints such as the shoulders, ankles, and knees substantiates a geriatric-specific vulnerability profile. This points to an associative kinematic pattern where weakened lower-extremity stability is frequently accompanied by compensatory, high-risk movements in the upper extremities.

Third, there is an urgent need for precision prevention strategies based on the “Sports Paradox.” The highest-risk scenario—high-intensity exercise for three or more hours daily in the early morning—suggests that healthy habits may transition into severe trauma when coupled with biorhythm imbalances and accumulated neuromuscular fatigue. This implies that exercising beyond one’s physiological readiness in dedicated facilities can be associated with counterproductive results. In conclusion, ensuring the sustainability of physical activity in later life requires a paradigm shift toward “Precision Safety Management” based on data, moving beyond simple facility expansion or universal safety campaigns. The high-risk scenarios identified in this study provide a scientific foundation for developing customized prevention programs and safety guidelines that reflect the unique activity profiles of elderly participants.

## Figures and Tables

**Table 1:** Injury sites and response details based on the survey.

No	Variable	Sub Variable									
1	Gender	Male					Female				
2	Injury Experience	Injured					Non-injured				
3	Participation Frequency	Daily		4–5 times a week			2–3 times a week			Once a week	
		2–3 times a month		Once a month			Once every 2–3 months			Once every 4–5 months	
		Once every 6 months		Irregularly							
4	Duration per Session	Less than 30 min			Less than 1 h				Less than 2 h		
		Less than 3 h			3 h or more						
5	Time of Activity	Early Morning (07:00–09:00)		Morning (09:00–12:00)			Lunchtime (12:00–15:00)			Afternoon (15:00–18:00)	
		Evening (18:00–21:00)			Night (21:00–24:00)				Late Night (00:00–05:00)		
6	Type of Facility	School Playground			Multi-purpose Sports Complex				Sport-specific Facility		
		Non-sport-specific Location			Other						
7	Injury Site	Scalp	Cheek			Upper Arm		Hip Joint			Ankle
		Eyes	Head			Elbow		Buttocks			Sole
		Nose	Neck			Forearm		Thigh			Instep
		Mouth	Chest			Wrist		Groin			Toe
		Teeth	Abdomen			Palm		Knee			Toenail
		Ears	Back			Back of Hand		Shin			Other
		Forehead	Waist			Finger		Calf			
		Jaw	Shoulder			Fingernail		Achilles Tendon			
8	Cause of Injury	Collision with another person		Struck by a ball			Tripped over an object			Excessive movement	
		Collision with facility		Collision with vehicle			Fall from height			Other	
		Struck by or collision with sports equipment			Slip and fall				Awkward landing after jump		

**Table 2:** Analysis of physical activity participation patterns among older adults (Top 5 rules by Lift).

Antecedents	Consequents	Support	Confidence	Lift
Late Night	Non-sport-specific Location, Irregular Participation	0.010	0.500	22.00
Sport-specific Facility, Less than 3 h	Once every 4–5 months	0.010	0.091	16.00
Less than 2 h, 2–3 times a week	Morning, Afternoon	0.011	0.182	12.80
3 h or more	Daily, Afternoon	0.010	0.061	10.667
Early Morning	Once a month, School Playground	0.010	0.100	7.040

Note. Dedicated facilities include standardized environments such as public gyms, swimming pools, and senior sports centers. Non-sport-specific locations include non-standardized environments such as public parks, mountains, or neighborhood roads.

**Table 3:** Analysis of physical activity participation patterns among male older adults (Top 5 rules by Lift).

Antecedents	Consequents	Support	Confidence	Lift
Less than 2 h, 2–3 times a week	Morning, Afternoon	0.011	0.188	12.75
Early Morning, Evening	Non-sport-specific Location, Less than 1 h	0.010	1.000	12.36
Once every 6 months	Afternoon, School Playground	0.011	0.375	11.33
Irregularly	Multi-purpose Sports Complex, Less than 30 min	0.010	0.095	8.63
Morning, Lunchtime	Sport-specific Facility, Once every 2–3 months	0.010	0.500	6.18

Note. Dedicated facilities include standardized environments such as public gyms, swimming pools, and senior sports centers. Non-sport-specific locations include non-standardized environments such as public parks, mountains, or neighborhood roads.

**Table 4:** Analysis of physical activity participation patterns among female older adults (Top 5 rules by Lift).

Antecedents	Consequents	Support	Confidence	Lift
Irregularly, Less than 30 min	Early Morning, Morning, Afternoon	0.013	1.000	80.00
Once a week, Less than 3 h	Morning, Afternoon, Evening	0.013	1.000	80.00
Early Morning, Night	4–5 times a week, Non-sport-specific Location	0.013	1.000	80.00
Once every 2–3 months	Sport-specific Facility, Early Morning, Morning, Afternoon	0.013	0.500	40.00
Early Morning	Multi-purpose Sports Complex, Daily	0.013	0.333	26.67

Note. Dedicated facilities include standardized environments such as public gyms, swimming pools, and senior sports centers. Non-sport-specific locations include non-standardized environments such as public parks, mountains, or neighborhood roads.

**Table 5:** Frequency analysis of injury causes among elderly grassroots sports participants.

No	Cause of Injury	N	%	No	Cause of Injury	N	%
1	Excessive movement	52	33.1	7	Struck by a ball	6	3.8
2	Slip and fall	34	21.7	8	Collision with facility	6	3.8
3	Collision with another person	15	9.6	9	Fall from height	3	1.9
4	Tripping over an object	14	8.9	9	Collision with vehicle/bicycle	3	1.9
5	Awkward landing after a jump	11	7	11	Other	2	1.3
6	Collision with sports equipment	9	5.7				

**Table 6:** Frequency analysis of injury sites among elderly grassroots sports participants.

No	Injury Site	N	%	No	Injury Site	N	%	No	Injury Site	N	%
1	Shoulder	11	11.6	11	Fingers	3	3.2	21	Groin	1	1.1
2	Ankle	11	11.6	12	Toenails	2	2.1	22	Cheek	1	1.1
3	Knee	9	9.5	13	Jaw	2	2.1	23	Ear	1	1.1
4	Elbow	9	9.5	14	Hip joint	2	2.1	24	Calf	1	1.1
5	Wrist	7	7.4	15	Sole	2	2.1	25	Toes	1	1.1
6	Shin	5	5.3	16	Forearm	2	2.1	26	Scalp	1	1.1
7	Lower back	5	5.3	17	Back of the hand	2	2.1	27	Chest	1	1.1
8	Other	4	4.2	18	Upper arm	1	1.1	28	Thigh	1	1.1
9	Achilles tendon	3	3.2	19	Buttocks	1	1.1	29	Mouth	1	1.1
10	Palm	3	3.2	20	Back of the foot	1	1.1	30	Teeth	1	1.1

**Table 7:** Analysis of sports injury patterns among elderly participants (Top 3 rules by Lift).

Antecedents	Consequents	Support	Confidence	Lift
Early morning, Excessive movement, Dedicated sports facilities, 3 h or more	Daily	0.017	1.000	7.43
Afternoon, 4–5 times a week, Excessive movement, Dedicated sports facilities	Less than 2 h	0.017	1.000	2.83
Morning, 4–5 times a week, Excessive movement, Dedicated sports facilities	Less than 2 h	0.017	0.667	1.88

Note. Dedicated facilities include standardized environments such as public gyms, swimming pools, and senior sports centers. Non-sport-specific locations include non-standardized environments such as public parks, mountains, or neighborhood roads.

## Data Availability

The datasets analyzed in this study were obtained from the official website of the Korea Sports Safety Foundation (KSSF, https://www.sportsafety.or.kr (accessed on 15 August 2025)). The data are publicly available for research purposes, and all raw data used in this study can be accessed through the foundation’s official data portal.
